# Automatic determination of the arterial input function in dynamic susceptibility contrast MRI: comparison of different reproducible clustering algorithms

**DOI:** 10.1007/s00234-015-1493-9

**Published:** 2015-01-30

**Authors:** Jiandong Yin, Jiawen Yang, Qiyong Guo

**Affiliations:** Department of Radiology, Shengjing Hospital of China Medical University, No. 36, Sanhao Street, Heping District, Shenyang, 110004 People’s Republic of China

**Keywords:** Affine propagation, Arterial input function, Cerebral perfusion, Normalized cut

## Abstract

**Introduction:**

Arterial input function (AIF) plays an important role in the quantification of cerebral hemodynamics. The purpose of this study was to select the best reproducible clustering method for AIF detection by comparing three algorithms reported previously in terms of detection accuracy and computational complexity.

**Methods:**

First, three reproducible clustering methods, normalized cut (Ncut), hierarchy (HIER), and fast affine propagation (FastAP), were applied independently to simulated data which contained the true AIF. Next, a clinical verification was performed where 42 subjects participated in dynamic susceptibility contrast MRI (DSC-MRI) scanning. The manual AIF and AIFs based on the different algorithms were obtained. The performance of each algorithm was evaluated based on shape parameters of the estimated AIFs and the true or manual AIF. Moreover, the execution time of each algorithm was recorded to determine the algorithm that operated more rapidly in clinical practice.

**Results:**

In terms of the detection accuracy, Ncut and HIER method produced similar AIF detection results, which were closer to the expected AIF and more accurate than those obtained using FastAP method; in terms of the computational efficiency, the Ncut method required the shortest execution time.

**Conclusion:**

Ncut clustering appears promising because it facilitates the automatic and robust determination of AIF with high accuracy and efficiency.

## Introduction

In healthy individuals, alterations in neural activity are accompanied by changes in local cerebral blood flow (CBF). Thus, as Logothetis NK pointed out, CBF has been used as a marker of neural activity in cognitive studies [1]. Moreover, CBF plays an important part in the diagnosis and management of several pathologies [2]. For example, Rau MK et al. reported that dynamic susceptibility contrast (DSC)-based cerebral blood hemodynamics can be used to predict tumor recurrence and survival time in those patients with gliomas [3]. Recently, Furtner J et al. demonstrated that quantified maximum tumor blood flow (mTBF) values could offer a new and totally noninvasive marker to prognosticate the event-free survival (EFS), independently on histopathological tumor grading, in patients with gliomas [4]. Therefore, cerebral perfusion is linked intimately to normal and abnormal brain functions, which is why numerous efforts have been made to quantify the CBF. For some urgent cases, it is also vitally important to obtain hemodynamic information rapidly.

To minimize overexposure to ionizing radiation from some imaging devices, such as xenon inhalation combined with computerized tomography and positron emission tomography, DSC-MRI has become a common imaging technique for cerebral perfusion, which relies on the intravenous injection of a contrast agent and the rapid measurement of transient MRI signal changes during the passage of the bolus through the brain [5].

Normally, the arterial input function (AIF) can be obtained from the mean curve of the bolus time-concentration curves that correspond to arterial pixels. Traditional methods for AIF extraction require operators to draw a region of interest on a large artery that passes through the imaged slice, such as the middle cerebral artery (MCA) and internal carotid artery [6]. However, the manual procedure is time-consuming and unrepeatable because it is based on operator’s experience and subjective judgment, which may have adverse effects on estimation of hemodynamic parameters [7, 8]. Automatic method for AIF detection is very attractive because it is much faster, less user-dependent, and more reproducible. Thus, some automatic methods have been developed. These methods include a class of techniques based on the cluster analysis of various multivariate statistical principles, such as fuzzy *c*-means (FCM), *k*-means, and hierarchy (HIER) algorithms [2, 7, 9]. Because of their high susceptibility to the randomly selected initial centers of the clusters, both FCM and *k*-means reduce the calculation-recalculation reproducibility of AIFs, and further result in highly detrimental effects on disease diagnosis and tracing, so the feasibility of using these two methods for AIF detection is doubtful [10]. Compared with FCM and *k*-means, the HIER method can generate reproducible result but it is highly time-consuming. Another clustering method reported by Frey and Dueck, fast affine propagation (FastAP), was applied to dynamic contrast-enhanced MRI (DCE-MRI) data to obtain the AIF, and it yielded absolutely stable results [10, 11]. However, the performance of this novel method has not been evaluated for AIF detection using DSC-MRI data. In addition, another more encouraging clustering method called the normalized cut (Ncut) algorithm is available, which uses an unbiased measure of the disassociation between different clusters [12]. It presents a good quality when minimizing the disassociation of different clusters, and it leads directly to maximizing the total association within the clusters [12].

In our opinion, the clustering methods reported previously for AIF detection can be divided into two kinds, reproducible algorithms (Ncut, HIER, and FastAP) and irreproducible algorithms (FCM and *k*-means). Recently, we compared two irreproducible methods (*k*-means and FCM) [13], and we also conducted a comparison between a reproducible method (Ncut clustering) and two irreproducible methods (*k*-means and FCM) [14]. However, to the best of our knowledge, there have been no comparisons between the reproducible clustering methods. Hence, it is still a question which reproducible algorithm can obtain more accurate and rapid results. We think it is necessary and important to solve this problem for clinical practice.

To address that problem, we compared the three reproducible clustering methods previously used for AIF detection in terms of the computational speed and detection accuracy. It was performed (1) on simulated data where the true AIF was known and (2) on DSC-MRI data obtained from 42 subjects where the AIF by manual drawing was extracted. To assess the performance of different methods to detect the AIF, we compared shape parameters of the estimated AIFs with the reference AIFs and also compared the computational time of the three algorithms. We think that this investigation is very important because a perfusion imaging method with improved accuracy and speed would be beneficial for clinical use.

## Methods

All of the experiments were performed using an off-line personal computer (Inter Core i3 M350 CPU processor, 2.27-GHz operating frequency, 2.0-GB RAM memory capacity, Microsoft Windows 7 operating system). A program based on MATLAB (R2010b) was developed in our department for AIF detection.

### PWI data acquisition

1. Simulated data

Normally, the first passage of AIF presents the following shape characteristics: it is a relatively flat trend at the beginning, then, there appears a rapid shape rise to a maximum concentration, and finally, we can see a slower decrease after the peak. The first passage does not return to the baseline value, but it generally overlaps with the smaller and wider peak of the second recirculation contribution, mainly from the part of the original injected bolus that was distributed to other organs, including the thyroid, kidneys, and lymph nodes [15]. The simulation was performed according to published methods [7, 9, 16]. First, the first passage of AIF was modeled as a gamma-variate function, as follows:

1 $$ \mathrm{A}\mathrm{I}\mathrm{F}(t)\propto \left\{\begin{array}{l}{\left(t-{t}_0\right)}^{\alpha}\times {e}^{-\left(t-{t}_0\right)/\beta}\kern1.25em t>{t}_0\\ {}0\kern7.75em t\le {t}_0\end{array}\right., $$

where *t*_0_ denotes the bolus arrival time and *α* and *β* are shape parameters that depend on the vasculature architecture and blood flow, respectively. Next, a recirculation was added that comprised the aforementioned AIF with a delay of *τ*_*d*_, which was convolved with an exponential time constant *τ*_*r*_. Based on previous studies, the parameters were set to *α* = 3.0, *β* = 1.5, *τ*_*d*_ = 8 *s*, and *τ*_*r*_ = 30 s [7, 16]. *t*_0_ was set to 26 s which closely approximates the arrival time of contrast agent for our clinical perfusion data.

According to the indicator dilution theory for intravascular contrast agents, the time-concentration curve of the contrast agent in the tissue voxel of interest, *C*_*t*_(*t*), is calculated using the following formula:

2 $$ {C}_t(t)=\mathrm{C}\mathrm{B}\mathrm{F}\times \mathrm{A}\mathrm{I}\mathrm{F}(t)\otimes R(t), $$

where the residue function *R*(*t*) is modeled as the following gamma-variate function.

3 $$ R(t)={e}^{\left(-\frac{t}{\mathrm{MTT}}\right)}, $$

where MTT is the mean transit time, which equals the ratio of the cerebral blood volume (CBV) relative to the CBF. Ideally, the bolus is injected instantaneously and subsequently washed out by perfusion.

Next, the MRI signal intensity at time *t*, *S*(*t*), is obtained by

4 $$ S(t)={S}_0\times \exp \left(-{\kappa}_{\mathrm{vox}}\times \mathrm{T}\mathrm{E}\times {C}_t(t)\right), $$

where *S*_0_ is the baseline signal intensity of the image, which was 100 in this study, and *κ*_vox_ is selected to produce a 40 % signal peak decrease from the baseline for normal gray matter (GM), i.e., the values found in typical clinical cases [17]. The time-intensity curves of the simulated MRI signal were generated according to the imaging form used in the clinical study mentioned below (frames, 60; echo time (TE), 0.03 s; repetition time (TR), 1.5 s).

Based on Peruzzo [9], the simulated data comprised six types of components, i.e.:

- Six “true” arterial voxels, i.e., not affected by the partial volume effect (PVE)
- Sixteen “false” arterial voxels
- Four hundred forty voxels that simulated normal GM tissue
- Four hundred forty voxels that simulated pathological GM tissue
- Six hundred voxels that simulated normal white matter (WM) tissue
- Four hundred voxels contaminated by PVE

These tissue states were simulated as follows [9], i.e., normal GM: CBV = 4 ml/100 g, MTT = 4 ± 0.33 s; pathological GM: CBV = 3.3 ml/100 g, MTT = 10 ± 0.7 s; and normal WM: CBV = 2 ml/100 g, MTT = 5.45 ± 0.33 s. Sixteen false arterial voxels were simulated by varying *t*_0_ from 27 to 30 s and *τ*_*d*_ from 9 to 12 s in increments of 1.0 s. The voxels contaminated by PVE were simulated using linear combinations of a true arterial signal and a signal for one of the different tissues, where the weights were selected at random.

Finally, 400 curves were extracted randomly from the simulated data and zero-mean Gaussian noise was added. The signal-to-noise ratio (SNR) (given by: SNR = *S*_0_/*σ*) was set to 20. In general, SNR = 20 is considered to be the typical noise level in clinical MRI data [9, 16, 18].

2. Human data

Ethical clearance for this study was obtained from the Ethics Committee at Shengjing Hospital, which is part of China Medical University (No. 2013PS113K), and written informed consent was obtained from each participant after a detailed explanation of the purpose of the study and scanning procedures.

In total, 42 healthy volunteers participated in this study (age = 23–69 years; average age = 49.5 years; weight = 58 ± 14 kg; 27 males and 15 females). The DSC-MRI data were acquired using a 3.0T whole-body MR scanner with multichannel capabilities (MAGNETOM Verio; Siemens Medical Solution, Erlangen, Germany). A single-shot echo planar imaging (EPI) sequence was used for perfusion imaging with the following parameters: TR = 1500 ms, TE = 30 ms, matrix = 128 × 128, field of view (FOV) = 23 × 23 cm, slice thickness = 4 mm, spacing between slices = 5.2 mm, slice number = 19, acquisition type = 2D, number of phase encoding steps = 127, transmitting coil = body, and flip angle = 90°. At the seventh time point, a gadolinium-based contrast agent (Gadovist; Bayer Schering Pharma AG, Berlin, Germany) was administered using an automatic power injector at a rate of 4 ml/s, followed by an equal volume of saline flush at the same injection speed. The temporal resolution (interval between two adjacent frames) was 1500 ms [19]. The horizontal part of the right MCA was covered by an imaging slice. Sixty-two whole-head images were obtained (scanning duration = 93 s) per subject. The magnetization state was not steady at the beginning of perfusion scanning, so the first two images were discarded. Therefore, 60 volumes were used for subsequent analysis.

### AIF determination

1. AIF calculation for simulated data

First, the intensity curves of the signal were converted into contrast agent concentration curves using the following equation [6, 7, 20, 21]:

5 $$ {C}_t(t)=-\frac{1}{\mathrm{TE}\times {\kappa}_{\mathrm{vox}}}\times \ln \left(\frac{S(t)}{S_b}\right), $$

where *S*_*b*_ is the pre-contrast (baseline) signal intensity, which is obtained by averaging the pre-contrast signal (i.e., *t* < 26 s).

Next, based on the mathematical principles outlined by Peruzzo et al., Shi et al., and Shi et al. [9, 10, 12], the FastAP, HIER, and Ncut methods were applied independently to the converted data (using Euclidean distance). The number of voxels is the number of samples for clustering. Based on previous studies, the clustering number was set to 5 [2, 9]. Normally, the time-concentration curves of the boluses in the arteries are characterized by a higher maximum concentration, an earlier maximum concentration, and a narrower full width half-maximum (FWHM), which allow the arterial curves to be distinguished from venous curves that appear wider with later bolus arrival, and tissue curves that are wider with a lower peak height [2, 10]. Thus, to determine the AIF automatically, several parameters were calculated for the mean curve of each cluster, including the maximum concentration (peak value, PV), time of maximum concentration (time to peak, TTP), and FWHM, as well as a measure (*M*) given by PV/(TTP × FWHM) [2]. The cluster with the maximum *M* value was considered as arterial pixels and the AIF was obtained from this mean curve.

2. AIF calculations using human data

First, due to the misalignments of the volume images at different time points caused by breathing, heartbeats, and the involuntary motions of the subjects, all of the volume images were aligned to the first pre-contrast volume based on a rigid transformation using SPM (available at http://www.fil.ion.ucl.ac.uk/spm/; version SPM99) and INRIAlign 1.01 (available at http://www-sop.inria.fr/epidaure/Collaborations/IRMf/INRIAlign.html) [22, 23]. We did not perform the smoothing operation for any of the images.

Second, the slice that covered the horizontal part of the right MCA was identified manually from the first volume image to calculate the AIF. It has been demonstrated that the selection of this slice can result in less error than other slices during CBF quantification because of its size and its location close to brain tissue [9, 24]. As with the simulation study, the time-intensity curves of the signals were converted into contrast agent time-concentration curves using Eq. 5.

Third, the arterial voxels comprised a minority in the selected slice; thus, most of the pixels represented tissue voxels with small changes in signal intensity. Therefore, the area under each concentration curve (AUC) was calculated and the *P*_AUC_ percentage of the curves with the smallest areas were excluded [10].

Fourth, during perfusion imaging, some fluctuating curves were obtained because of shifts in voxels, PVEs, physiological pulsations, and other uncontrollable effects. These irregular curves would result in poor estimates of the true AIF. Thus, a standard measurement of curve smoothness was used, Eq. 6, and the *P*_rough_ percentage of the remaining curves with the largest integral values were excluded [2].

6 $$ \wedge (C)={\displaystyle {\int}_o^T{\left[{C}_t{\prime\prime} (t)\right]}^2\mathrm{d}t} $$

As reported by Mouridsen et al., the values of *P*_AUC_ and *P*_rough_ were predefined as 0.90 and 0.25, respectively [2].

Fifth, the novel method reported recently by Bleeker et al. was applied to the remaining curves to further reduce contamination by PVE, which used the ratio of the steady-state integral value relative to the AUC for the first passage [25]. This was simplified as follows. The first passage of the contrast agent was fitted to a gamma variate function, and the area under each fitted curve was calculated and abbreviated to AUC^1st^. The beginning of the steady state was defined as the first time point that was <30 % of the maximum after the peak of the time-concentration curve [21] and ten subsequent time points were integrated, which was abbreviated to SS. Finally, the mean ratio of SS to AUC^1st^ was calculated, and curves where the ratios fell outside the range of acceptance (mean ratio ± 20 %) were discarded.

As with the simulation study, the remaining curves were regarded as the input data and the Ncut, HIER, and FastAP methods were implemented independently. The number of clusters was still set to 5 and the AIF cluster was again determined using the measure *M* = PV/(TTP × FWHM).

Unlike the simulation study, no gold standard AIF was available for comparison to determine the clustering algorithm that yielded more accurate results. Thus, based on the method proposed by Shi et al. [10], the performance of each clustering method was evaluated by comparisons with the manual method. According to the method proposed by Mouridsen et al., six potential arterial voxels with the shape characteristics of an earlier TTP, higher PV, narrower FWHM, and quicker washout [2, 10] were located by an experienced neural radiologist (work experience, 34 years). The manual AIF was obtained by averaging these six time-concentration curves.

### Statistical analysis

1. Analysis of the simulation study

First, the parameters of the estimated AIFs were calculated to evaluate the AIF detection performance of the three different clustering methods [26]. The selected AIF clusters were potentially contaminated; thus, the PVE level was defined as the percentage of non-arterial signal in the AIF cluster, i.e., a lower PVE level meant that the corresponding clustering algorithm could discriminate the arterial regions well [9]. Moreover, based on Eq. 1, we can find that the shapes of the estimated AIFs would have profound effects on subsequent CBF quantification. Various parameters related to the shape feature were calculated, i.e., FWHM, TTP, and PV [26]. In fact, the true AIF has the shape characteristics of an earlier TTP, higher PV, and narrower FWHM [10].

Second, the relationship between the AIF integral and CBV is a linear correlation, so the area under the AIF curve (AUC) was also used to evaluate the accuracy of the estimated AIFs [9, 26].

Finally, the difference between the estimated AIF and the true AIF was computed as the root mean squared error (RMSE) [9]:

7 $$ \mathrm{RMSE}=\sqrt{\frac{{\displaystyle {\sum}_{i=1}^n{\left[\left({\mathrm{AIF}}_{\mathrm{estimate}}\left({t}_i\right)-{\mathrm{AIF}}_{\mathrm{true}}\left({t}_i\right)\right)\right]}^2}}{n}}, $$

where *n* is the scan time (90 s).

2. Analysis of the clinical study

The true AIF curve had the shape characteristics of an earlier TTP, narrower FWHM, and higher PV [10], so the feature parameters of the AIFs derived from different clustering methods were calculated in a similar manner to the simulation study. In addition, as reported by Shi et al. [10], the error was also assessed using Eq. 7, where the true AIF was replaced by the manual AIF. Moreover, to investigate the computational complexity, the execution time of each algorithm was recorded. The statistical analysis was performed using SPSS (SigmaStat, 2.03, Inc., Chicago, IL) with a *t* test, where *P* < 0.05 was considered to indicate a significant difference.

## Results

### Results based on the simulated data

**Fig. 1:**
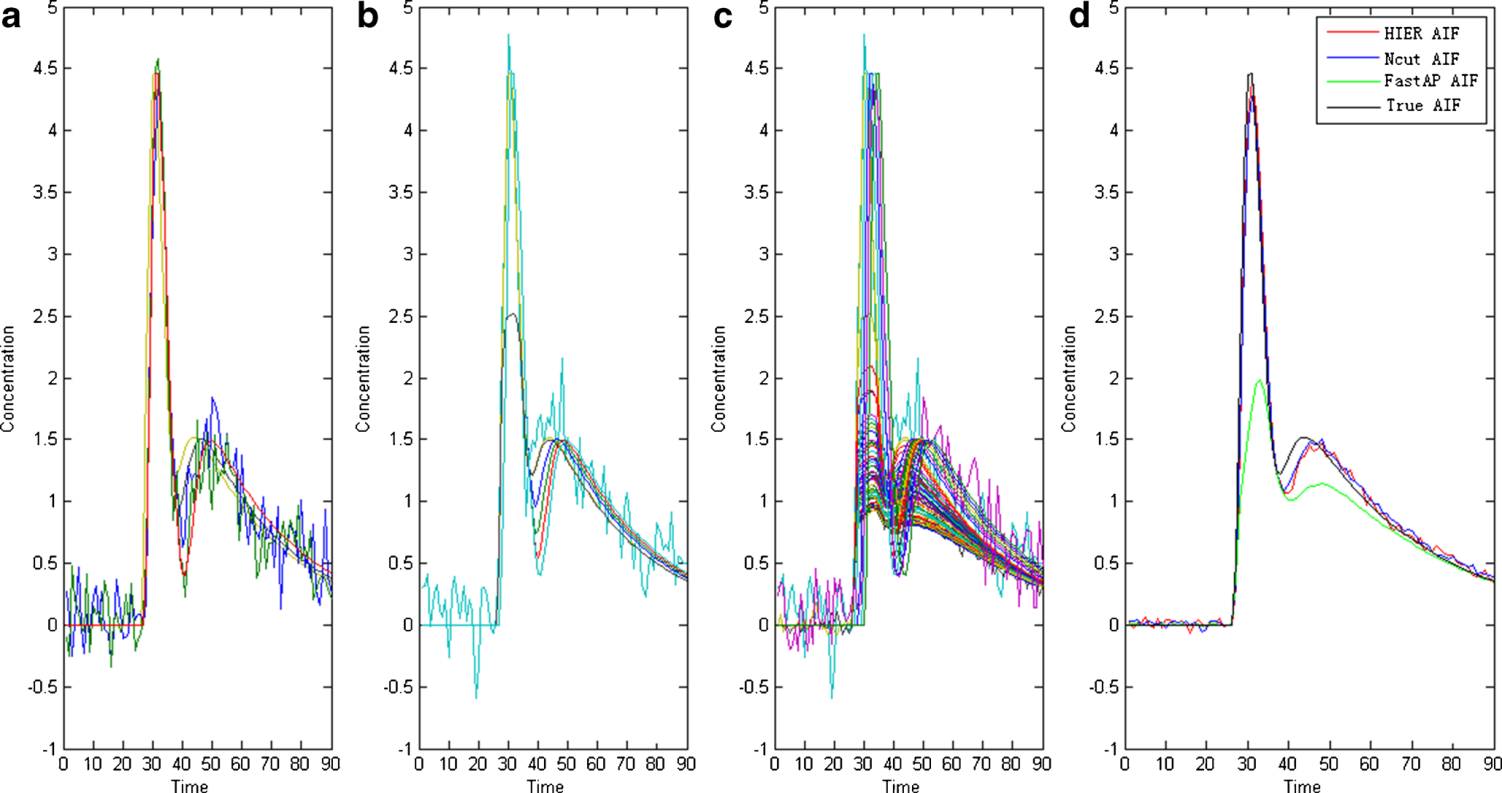
Results obtained using different clustering algorithms. **a**–**c** Arterial clusters obtained using the HIER, Ncut, and FastAP clustering methods, respectively, and **d** comparison of the AIFs obtained using different algorithms with the true AIF

The results are shown in Fig. 1 and Table 1.

**Table 1:** Comparison of the AIFs obtained from simulated data using different clustering methods and the true AIF

AIF	PVE	PV	TTP	FWHM	AUC	RMSE	*M* value
HIER-based AIF	40.00	4.4030	30.01	6.3188	76.2088	0.1374	0.0232
Ncut-based AIF	45.45	4.2737	29.92	6.4563	76.4836	0.0519	0.0221
FastAP-based AIF	92.41	1.9933	31.75	27.6158	57.3931	5.5088	0.0023
True AIF	0	4.4592	29.51	6.2016	76.8669	0	0.0244

Table 1 shows that the results obtained with the Ncut method were very similar to those with the HIER method, which is also confirmed by Fig. 1d. In contrast to the FastAP algorithm, the AIFs obtained using the other two clustering methods were characterized by a higher PV, earlier TTP, narrower FWHM, and larger AUC and *M* values; thus, they were closer to the true AIF. This means that the Ncut and HIER methods are more suitable for AIF detection. The lower AIF detection performance of FastAP might have been attributable to severe PVE contamination, which is supported by Fig. 1c. According to the RMSE indicator in Table 1, the Ncut-based AIF was generally more similar to the true AIF.

### Results based on the human data

**Fig. 2:**
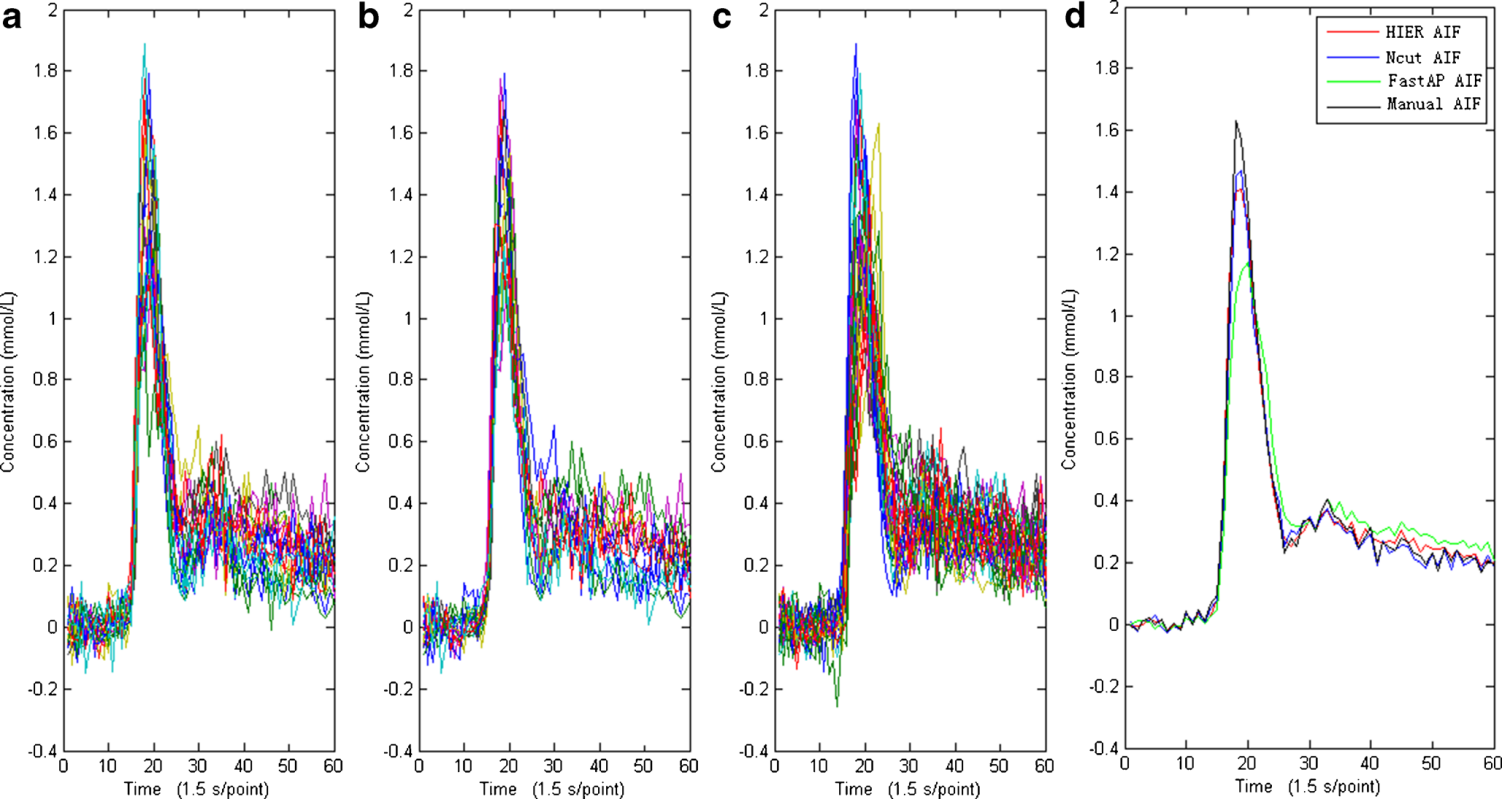
AIF detection results obtained using different clustering methods. **a**–**c** Arterial clusters extracted using the HIER, Ncut, and FastAP methods, respectively, and **d** comparison of the manual AIF and estimated AIFs based on different algorithms

**Fig. 3:**
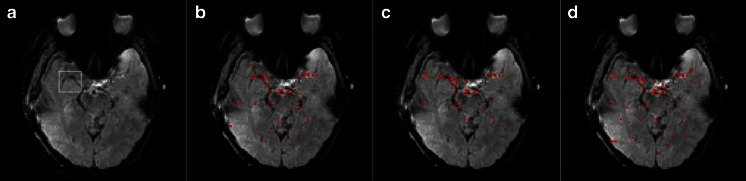
Detection results for the arterial regions based on different clustering methods. **a** Optimal slice image used for clustering analysis, where the *horizontal* part of the right MCA is indicated by the *white rectangle*, and **b**–**d** the AIF detection results obtained using HIER, Ncut, and FastAP, respectively, where the *red pixels* indicate the arterial regions. Compared with the FastAP algorithm, the Ncut and HIER methods could obtain more accurate arterial regions, and they increased the AIF detection performance

The human AIFs were determined using the different clustering methods. A randomly selected human subject was used to illustrate the differences in the AIF detection results obtained with the three clustering methods (Figs. 2 and 3, Table 2). Figure 2 shows that the results obtained using the human data presented similar manner to those in the simulation study. First, the HIER- and Ncut-based AIFs had similar shape characteristics, which were closer to the manual AIF results than the FastAP-based AIF results. Second, the HIER- and Ncut-based AIF results had higher peaks than the FastAP-based AIF results. As noted by Enmi et al. [27], these results demonstrated that the HIER and Ncut methods were affected less by PVE contamination during AIF detection. This viewpoint can be appreciated intuitively based on Fig. 2c. Third, according to the quantitative parameters of the shape characteristics shown in Table 2, i.e., the PV, TTP, FWHM, AUC, *M* value, and error, we found that the Ncut method yielded the best AIF results based on the human data, which agreed with the conclusion of the simulation study. Based on Fig. 3, we can intuitively come to the conclusion that there were only minor differences as shown. We also found that most of the arterial regions near the MCA were detected in the tissue rather than in voxels that passed through the arteries. This finding was counterintuitive, but it agreed with the results reported by Bleeker et al., where the best AIF measurements were also obtained from voxels located in the tissue surrounding the MCA [28].

**Table 2:** The parameters of AIF detection results based on different methods for the randomly selected subject

AIF	Shape parameters			AUC	*M* value	Error	Execution time (s)
	PV (mmol/l)	TTP(s)	FWHM(s)				
HIER-based AIF	1.4239	17.53	6.0285	18.8842	0.0135	0.03489	428.4631
Ncut-based AIF	1.5167	17.50	5.6819	19.1716	0.0153	0.03118	0.4183
FastAP-based AIF	1.187	18.80	7.3869	17.6263	0.0085	0.09036	29.9891
Manual AIF	1.6703	17.34	5.3394	19.6311	0.0180	0	107.3476

For 42 volunteers, the pattern of differences in the AIF shape parameters with the three methods was similar to those observed in the simulation study. The detailed results of the statistical analyses are shown in Table 3. Similar to the results of the simulation study, the AIFs based on the Ncut algorithm had a higher PV, similar TTP, narrower FWHM, larger AUC and *M* values, and smaller error values. There were significant differences in the PV, FWHM, and AUC between the Ncut and FastAP methods (all *P* values <0.05) but not between the Ncut and HIER methods (all *P* values >0.05). There was no significant difference in the TTP between the Ncut and HIER methods or between the Ncut and FastAP methods (all *P* values >0.05). In terms of the execution time, *M* values, and error values, the differences between Ncut and each of the other two methods reached significant level (all *P* values <0.05).

**Table 3:** Comparison of the AIF detection results obtained from clinical data using different clustering methods

AIF	Shape parameters			AUC	*M* value	Error	Execution time (s)
	PV (mmol/l)	TTP (s)	FWHM (s)				
HIER-based AIF	1.6896 ± 0.1768	30.55 ± 1.09	6.3165 ± 0.8846	18.9324 ± 1.2356	0.0142 ± 0.0011	0.0493 ± 0.0094	393.4381 ± 68.3683
Ncut-based AIF	1.7395 ± 0.1728	30.95 ± 0.36	5.5923 ± 0.2935	19.1081 ± 2.6085	0.0182 ± 0.0031	0.0397 ± 0.0076	0.4406 ± 0.1003
FastAP-based AIF	1.3977 ± 0.3264	31.98 ± 0.72	7.8908 ± 1.3431	17.8925 ± 3.2914	0.0076 ± 0.0015	0.0846 ± 0.0113	21.3792 ± 4.1271

## Discussion

AIF plays an important role in the quantification of cerebral perfusion using the DSC-MRI technique. The traditional manual method for AIF selection is subjective, which means that the results lack accuracy and reproducibility between different operators, as well as between different time points. Moreover, for some urgent cases, the AIF cannot be obtained sufficiently by rapidly using the manual procedure to make a timely decision [6]. Thus, the time-consuming nature of the manual method also prevents its wide application in clinical practice. The results obtained using multivariate statistical analyses based on traditional *k*-means and FCM clustering algorithms are highly sensitive to the initially selected cluster centers, thereby yielding unrepeatable results [9]. Thus, they are highly disadvantageous for the accurate diagnosis of patients and progression tracking. Therefore, it is necessary to develop a more robust, accurate, and rapid method for automatic AIF detection.

In this study, we evaluated the AIF detection performances of three previously reported clustering methods, Ncut, FastAP, and HIER. In contrast to traditional *k*-means and FCM, all of the algorithms used in the present study are known to be robust. The results of our simulation study and clinical study demonstrated that compared with the FastAP method, the Ncut and HIER algorithms yielded AIFs more in line with the expected AIF, with a higher PV, earlier TTP, narrower FWHM, larger AUC and *M* value, and smaller RMSE. The results demonstrated that the FastAP method delivered low AIF detection performance. In addition, according to the concept of “time is brain,” the time required to execute each algorithm was another important consideration in the present study. Of the two superior algorithms, the HIER method required far more time than the Ncut method and the difference was significant. For some urgent cases such as acute stroke, speed can save lives; thus, it is necessary to obtain the AIF results as soon as possible. Therefore, the Ncut method should be given priority for AIF detection in a clinical environment because of its higher detection accuracy and lower time consumption.

By definition, AIF should be measured from the small arterioles that supply blood to the corresponding tissue, such as M3 segment of middle cerebral artery (MCA). However, because of relatively coarse spatial resolution of DSC-MRI (the typical size is 2 × 2 × 5 mm^3^), a considerable PVE from the surrounding tissue would be produced, which could lead to major errors in the measurement of the shape of the AIF. In contrast, if we measure the AIF from a large artery, such as internal carotid artery (ICA), the measured AIF shape would be an erroneous representation of the bolus ultimately entering the tissue. Hence, in practice, a medium size of artery, such as M1 segment of MCA, is commonly chosen as a compromise between minimizing PVE and bolus delay and dispersion [6].

It must be emphasized that there were three limitations in the present study. First, only 42 subjects participated in the clinical study and this limited number of cases might have resulted in statistical uncertainty. Thus, it will be necessary to increase the number of subjects in a future study. Second, we only calculated the global AIF. The arterial dispersion of the bolus between the site where the AIF was measured and the true AIF could have led to an underestimation of the AIF [29]. To address this problem, the AIF of each pixel needs to be determined, i.e., the local AIF. Only a few attempts have been made previously to detect the local AIF because of the increased risk of PVE contamination and a reduced SNR [29–33]. Many different deconvolution approaches are available, however, which reduce the impact of tracer delay and dispersion, including time-insensitive block-circulant singular value decomposition and nonlinear stochastic regularization [33–38]. Third, all of the subjects who participated in this study were healthy. Thus, the clinical utility was not fully validated in patients with neurological diseases, such as acute stroke, arterial stenosis, and other abnormalities.

## Conclusion

In conclusion, we tested the suitability of three robust clustering algorithms for AIF detection based on a simulation study and clinical verification. Ncut algorithm appears to be highly promising because it facilitates automatic and robust AIF detection with high accuracy and low computational complexity.
